# Efficacy of Disinfectants for Monkeypox Virus Inactivation on High Touch Surface Materials in Low-Resource Settings

**DOI:** 10.1021/acs.est.4c09821

**Published:** 2024-10-31

**Authors:** Ana K. Pitol, Siobhan Richards, Patrick Mirindi, Hibak O. Mahamed, April Baller, Grant L. Hughes, Sara E. Beck

**Affiliations:** 1 Departments of Vector Biology and Tropical Disease Biology, Centre for Neglected Tropical Diseases, Liverpool School of Tropical Medicine, L3 5QA Liverpool, U.K.; 2 Department of Civil Engineering, University of British Columbia, 2002-6250 Applied Science Lane, Vancouver, British Columbia V6T 1Z4, Canada; 3 Infection Prevention and Control (IPC) and Water, Sanitation and Hygiene (WASH) Team, Country Readiness Strengthening (CRS) Department, WHO Health Emergencies (WHE) Programme, World Health Organization, 1211 Geneva, Switzerland

**Keywords:** mpox, orthopoxvirus, fomites, surface disinfection, ethanol, hydrogen peroxide, quaternary ammonium compounds, sodium hypochlorite, healthcare-associated infection

## Abstract

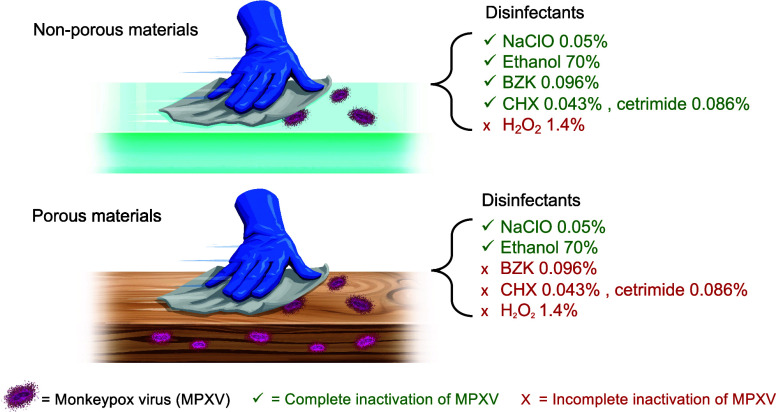

Disinfection efficacy tests were conducted on surface carriers inoculated with the monkeypox virus (MPXV) by applying six disinfectant solutions (and three controls) on six surfaces common in low-resource settings: four nonporous surfaces (stainless steel, glass, plastic, and latex) and two porous surfaces (ceramic and wood). Disinfectants were wiped on carriers in triplicate, with a 1 min contact time: 0.05 and 0.5% sodium hypochlorite, 70% ethanol, two quaternary ammonium compound (QAC)-based disinfectants, and 1.4% hydrogen peroxide. MPXV was then quantified, and log_10_ removal values were calculated. Sodium hypochlorite (0.05 and 0.5%) and ethanol (70%) removed MPXV to below detection level, ≥ 99.97% reduction for nonporous surfaces, and ≥99.40% for wood, QAC-based disinfectants were efficacious on nonporous surfaces (≥99.97% inactivation) but had diminished efficacy on wood, a porous surface, and 1.4% H_2_O_2_ had limited efficacy across all tested surfaces. Results varied by disinfectant type and surface type. Based on our results, we recommend using 0.05% sodium hypochlorite or 70% ethanol with 1 min contact time to inactive MPXV on clean nonporous and porous surfaces. As MPXV is evolving, future research with additional disinfectants, application methods, and environmental conditions and research to understand adsorption, disinfection efficacy, and transmission risk on porous surfaces are needed to develop practical disinfection recommendations.

## Abstract 2

This study evaluated the efficacy of surface disinfection on monkeypox virus (MPXV). Six disinfectant solutions were applied onto six surface materials relevant to low resource contexts using a real-world cleaning practice of wiping with a microfiber cloth saturated with disinfectant.

## Introduction

The recent declaration of the mpox outbreak as a public health emergency of international concern (PHEIC) highlights the need for interventions to interrupt transmission, including transmission via surfaces. Mpox, formerly known as monkeypox, is a viral disease endemic to forested regions of West and Central Africa. However, during the multicountry outbreak in 2022–2023, rapid geographic expansion occurred across historically nonendemic regions. Since January 2022, mpox has spread to at least 123 countries worldwide.^1,2^ Mpox is a viral disease caused by the monkeypox virus (MPXV), a member of the Orthopoxvirus genus, which also includes variola virus and vaccinia virus.^3^ MPXV is evolving and is currently divided into two genetic clades (Clade I, II), and four subclades (Ia, Ib, IIa, IIb). Clade I is potentially more virulent than Clade II.^4^ MPXV can be transmitted to humans through direct contact (also described as close contact) with infected lesions, bodily fluids, or respiratory secretions from the infected hosts, indirect contact through contaminated surfaces or materials (fomites), and through contact with infected animals.^5−7^ For the ongoing 2022–2024 outbreak of mpox (subclade IIb), sexual contact has been the primary transmission route.^8^ A new clade of mpox, Clade Ib, emerged in September 2023 in Democratic Republic of Congo and since spread to multiple countries in Africa.^9^

Healthcare-associated infections and community transmission of MPXV have been documented in various endemic and nonendemic countries.^6,10−12^ Fomites are one transmission route for MPXV and MPXV DNA has been detected on surfaces and objects in hospitals where mpox patients were treated and in the homes of individuals infected with MPXV.^13−17^ Notably, infectious MPXV has been found on objects such as underwear, sink tabs and tables at concentrations as high as 3.2 PFU/sample (underwear) up to 15 days after patients had vacated the premises.^14,15^ These findings align with experimental studies evaluating the persistence of orthopoxviruses (MPXV and vaccinia virus) on surfaces, which demonstrate that poxviruses can survive for extended periods, from days to months, on both porous and nonporous surfaces under various environmental conditions.^18−22^ MPXV surface survival is influenced by environmental temperature and humidity, surface material, and the matrix used to inoculate the surface.^18,21^ For example, when MPXV suspended in blood and semen was dried on surfaces, the half-lives were 39 and 6 days, respectively; however, that persistence decreased to 0.1–0.2 days when the inoculation matrix was saliva, urine, or feces.^18^ This is consistent with other studies showing increased viral persistence when viruses are inoculated on surfaces using liquid matrices with high protein content.^18,23^ This observed presence of infectious MPXV on surfaces for extended periods underscores the importance of efficacious surface disinfection protocols.

There has been limited previous research on disinfection efficacy with the monkeypox virus (MPXV) on surface materials, particularly with surfaces and disinfectants relevant to low-resource contexts. While some studies have evaluated disinfectant efficacy against vaccinia virus,^24^ variola virus,^25^ and MPXV,^21,26^ most of these studies were suspension tests, where a virus inoculum is mixed with disinfectant in suspension. Suspension tests often yield more favorable results, indicating higher efficacy, compared to tests where the virus is applied to a surface and then disinfected.^27−30^ While both suspension and surface tests are valuable for assessing disinfectant efficacy, they serve different purposes and contexts. Suspension tests are often conducted first to establish efficacy, and are then followed by surface carrier tests, where a viral inoculum is applied to a surface and allowed to dry, and then a disinfectant is applied to the contaminated surface for a specified contact time before recovering the remaining virus.^31,32^ Although useful, suspension and surface carrier tests do not fully replicate real-world cleaning practices, such as wiping with a disinfectant-soaked cloth, which is recommended by the World Health Organization.^33^ Therefore, this study involved a more real-world cleaning practice using a microfiber cloth saturated with disinfectant, following a method similar to those described elsewhere.^34,35^

Disinfectants such as chlorine-based compounds, alcohols, and solutions containing hydrogen peroxide or quaternary ammonium compounds are commonly used against orthopoxviruses due to their availability and their broad-spectrum antimicrobial activity. However, their efficacy can vary widely.^24^ The pathogen targeted, the surface material, and the chemical composition of the disinfectant, its concentration, and contact time, play a critical role in disinfectant efficacy.^36−39^ Therefore, it is necessary to evaluate the efficacy of disinfectants against the virus of concern on relevant surface materials at adequate concentrations.

In this study, we evaluated the efficacy of disinfectants commonly used in countries where mpox is endemic, at recommended in-use concentrations, using a methodology that reflects real-world scenarios. We tested a range of commercially available disinfectants on various high-touch surface materials and provided a comprehensive analysis of their efficacy in a worst-case scenario laboratory experiment.

## Methods

### MPXV Propagation

MPXV (Isolate 2225/22 Slovenia ex Gran Canaria, clade IIb) was amplified using BHK-21 cells (Syrian Golden Hamster) maintained at 37 °C with 5% CO_2_ in Dulbecco’s Modified Eagle’s Medium (DMEM; Corning) supplemented with 10% Fetal Bovine Serum (FBS; Sigma-Aldrich) and 0.05 mg/mL gentamicin (Gibco). For infection, the BHK-21 cells were cultured in DMEM media supplemented with 2% FBS. To amplify MPXV, a T-150 flask of confluent BHK-21 cells was inoculated with 2 × 10^4^ Plaque Forming Units (PFU) and incubated for 5 days. Cytopathic effects were monitored daily throughout infection. At day five post infection, the media was recovered and centrifuged for 15 min at 5000 rpm to remove cells and debris. This MPXV stock solution (5 × 10^7^ PFU/mL) was aliquoted and stored at −80 °C until use. All experiments were conducted using infectious MPXV in Containment Level 3 laboratories at the Liverpool School of Tropical Medicine (LSTM) by trained and Invanex-vaccinated personnel using approved standard operating procedures.

### MPXV Enumeration

Infectious MPXV was enumerated using standard plaque assays with Vero E6 cells (African green monkey kidney cells, Public Health England). Vero cells were maintained at 37 °C with 5% CO_2_ in DMEM, supplemented with 10% FBS and 0.05 mg/mL of gentamicin. Plaque assays were performed as follows: samples were serially diluted, and the undiluted sample and subsequent dilutions were inoculated on 24-well plates containing a confluent monolayer of Vero E6 cells. One hour after infection, a 0.8% Methylcellulose and DMEM media overlay supplemented with 2% FBS was applied to the cell monolayer and incubated at 37 °C with 5% CO_2_ for 5 days. Following incubation, cells were fixed with Formalin 10% (VWR International) and stained with Crystal Violet (Sigma-Aldrich) before quantifying plaques. Each sample was plated once, and all experiments were performed in three independent replicates, conducted on separate days.

### Surface Disinfection

#### Surface Carriers

We assessed the efficacy of surface disinfectants against MPXV on six different surface materials. Five surfaces common in low-resource settings were tested, including three nonporous materials (plastic, latex, glass) and two porous materials (wood, ceramic). Nonporous stainless steel was also tested as a standard.^31,32^ Circular coupons (12.7 mm diameter) of ceramic tile, stainless steel, plastic (polyethylene terephthalate), and borosilicate glass (Biosurface Technologies) were autoclaved at 121 °C and 15 psi for 1 h for disinfection. Circular coupons of unvarnished wood (Olycraft, 15 mm diameter) and steel covered in latex (Shield Latex Gloves, 12.7 mm diameter) were disinfected by soaking in 70% Ethanol (VWR International) for 15 min. All coupons were then rinsed with sterile Milli-Q water and allowed to dry for 2+ hours in a Class II Biosafety Cabinet. Plastic, glass, ceramic, and stainless-steel coupons were reused after disinfection (as above), while wood and latex-covered coupons were disposable.

#### Surface Disinfection Formulations

We selected five disinfectants, based on their availability in low-resource settings as well as their potential efficacy for MPXV disinfection. One of these disinfectants was evaluated at two concentrations, creating a total of six disinfectant solutions: a 0.5% and 0.05% solution of sodium hypochlorite (NaClO, Reagecon), 1.4% solution of hydrogen peroxide (H_2_O_2_, Monicare), a quaternary ammonia compound (QAC) containing 0.096% benzalkonium chloride disinfectant (BKZ, Dettol), a common disinfectant containing two QAC’s (0.043% chlorhexidine gluconate (CHX) and 0.086% cetrimide) (Savlon), and 70% Ethanol (VWR International). Sodium hypochlorite solutions were prepared on the day of the experiment by diluting 5% w/v NaClO, respectively. Chlorine concentration in the 0.05% solution was confirmed before each experiment using Chlorine Test Strips (Serim Monitor for Chlorine 100–750 ppm).^40^ The remaining disinfectants were purchased commercially, unexpired, and stored at room temperature (22 °C).

#### Surface Disinfection with Wiping Method

To assess disinfectant efficacy, surface coupons were inoculated with a 10 μL droplet of 9 parts MPXV viral stock (∼5 × 10^7^ PFU/mL) and 1 part of interfering substance (Bovine Serum Albumin, BSA at 3g/L, Fisher Scientific), in the center of the coupon. The droplet was distributed evenly across the surface and allowed to dry for 60 min at room temperature. Coupons were then disinfected by wiping the surface one time with a 2 cm^2^ microfiber wipe (Sainsbury’s; 88% polyester, 12% polyamide) saturated with disinfectant. The average volume of water-based disinfectant absorbed by the wipe was 0.098 ± 0.014 mL/cm^2^. To control the pressure applied during wiping process, coupons were placed on a balance and the applied pressure was maintained between 80 and 120 g/cm^2^ (1.1–1.7 psi). One minute after wiping, coupons were placed in 12-well plates containing 500 μL of ice-cold recovery media (DMEM + 2% FBS) to neutralize any residual disinfectant on the coupon. To recover the virus from the coupon, the media was pipetted 20 times before being transferred to a cryotube for subsequent analysis. Samples were stored immediately at −80 °C and analyzed within 1 week. Samples were quantified using standard plaque assays as described above. A no-wipe control, dry wipe control, and wipe with distilled water control were run alongside each experiment. Temperature and humidity, which were recorded throughout the experiment, fluctuated between 21 and 22 °C and 30–50%, respectively. All tests were conducted in triplicate.

#### Product Neutralization and Cytotoxicity Assessment

Before evaluating the antiviral efficacy of testing products, it was crucial to validate the neutralization process and to assess potential cytotoxic effects that the disinfectants could have on the cells. To determine if the testing products were adequately neutralized using ice-cold media, we used a modified version of the standard methodologies BS EN 14476^31^ and ASTM E2967–15,^34^ adapted to fit the experimental protocol described above. Briefly, stainless steel coupons were inoculated with a 10 μL droplet of culture media (DMEM). The media was allowed to dry for 60 min at room temperature. Subsequently, the coupons were disinfected by wiping the surface once with a 2 cm^2^ microfiber wipe saturated with disinfectant. The disinfectant was left on the surface for 1 min and then neutralized by placing the coupons in 12-well plates containing 500 μL of ice-cold recovery media (DMEM + 2% FBS). After neutralization, samples were inoculated with 10 μL of 5 × 10^7^ PFU/mL of MPXV and incubated for 15 min. Samples were quantified using standard plaque assays as previously described. Comparable levels of infective MPXV were expected to be recovered from the control (no disinfection) and the neutralized test substance for the neutralization to be considered valid. To assess for possible cytotoxicity of the cells by the residual disinfectant on the coupons, we repeated the experiment described in the neutralizer validation without the addition of the viral inoculum. The samples were then serially diluted and plated, and cytotoxicity was observed 1, 2, and 5 days later.

#### Data Analysis

All experiments were conducted in triplicate. The viral concentrations in the control and treatment groups were measured and reported as PFU per surface, with all values log_10_ transformed (log_10_ PFU/surface) for analysis. For both control and treatment groups, the mean and standard deviation values were calculated by log transforming the data and subsequently estimating the descriptive measures. The log_10_ reduction (LR) in viral concentration following treatment was determined by subtracting the concentration of virus in the control group from the concentration in the treatment group.

## Results and Discussion

### Product Neutralization and Cytotoxicity Assessment

In the neutralization assay, we observed comparable infectious MPXV levels recovered from the no-treatment control and the samples containing test substances neutralized with ice-cold media (difference between the control and samples <0.2 log_10_ for all the disinfectants, see Table 1). This finding indicates the effective neutralization of the products evaluated in this study. Furthermore, no cytotoxicity was observed at any dilution, as demonstrated by the sustained viability of the cells across all evaluated time points 1-, 2-, and 5-days postinoculation.

**Table 1:** Product Neutralization Assay^a^

treatment	average concentration (log_10_ PFU/mL)	difference (|log_10_|)
virus titer	4.84 ± 0.03	
water	4.92 ± 0.03	0.07
70% ethanol	4.90 ± 0.09	0.05
0.5% NaClO	4.92 ± 0.08	0.08
0.096% BKZ	5.02 ± 0.03	0.17
1.4% H_2_O_2_	4.93 ± 0.07	0.09
0.043% CHX & 0.086% cetrimide	4.89 ± 0.08	0.05

a MPXV titer recovered after product neutralization (log_10_ PFU/mL) and difference between the virus titer in the control samples and the virus titer in the treatment samples (Abs log_10_). Data represents the average of three replicates for each treatment. Only the highest concentration of NaClO was evaluated in the neutralization assay.

**Figure 1:**
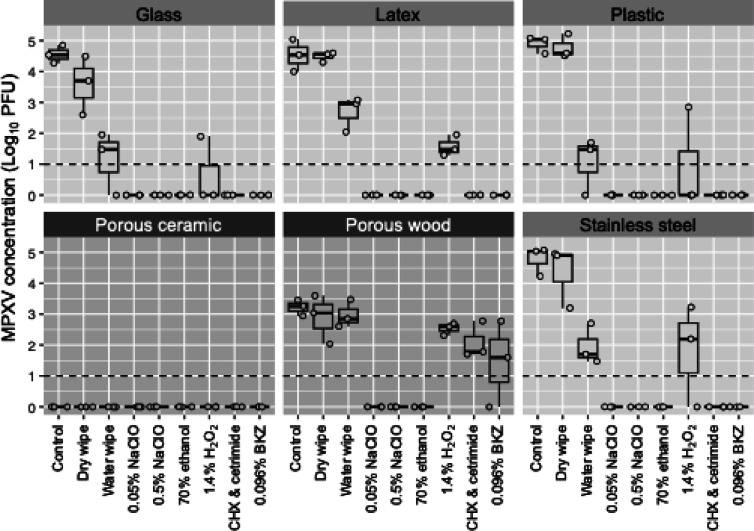
Postdisinfection recovery of monkeypox virus (MPXV) on six surfaces. Data represents the number of viruses (log_10_ PFU) recovered from various surfaces after wiping the surfaces with a dry wipe, water, or disinfectants. Porous surfaces (dark gray plots) include unvarnished wood and ceramic. Nonporous surfaces (light gray plots) include plastic, stainless steel, glass, and latex. Boxplots display the minimum, maximum, median, and interquartile range from three independent replicates. The LOD of the assay was 10 PFU (1 log_10_). Values below the LOD are visually represented as 0 in this figure.

We evaluated six different disinfectant solutions. on six different surfaces (four nonporous and two porous), using a methodology designed to replicate real-world wiping scenarios. The methodology utilized evaluated the disinfectants using a short, practical contact time (1 min) to create a worst-case disinfection scenario. On nonporous surfaces, wiping the surface with water alone reduced the number of MPXV recovered from the surface between 1.8 and 3.8 log_10_, depending on the surface. This reduction was similar to the reduction observed with H_2_O_2_ on some surfaces; however, the limited sample size (three replicates per condition) restricted our statistical analysis, preventing us from determining whether there was a statistically significant difference between the two conditions. Five of the six disinfectant solutions tested (0.5% NaClO, 0.05% NaClO, 70% ethanol, 0.096% BKZ, and 0.043% CHX with 0.086% cetrimide) reduced infectious MPXV in all replicates below the limit of detection (LOD, 10 PFU/surface), achieving an inactivation greater than 3.5 log_10_, or ≥99.97% reduction (Table 2). In contrast, the sixth disinfectant, 1.4% H_2_O_2,_ did not reduce surface MPXV concentrations below the LOD, where MPXV virus was recovered from at least one replicant on all surfaces with H_2_O_2_ treatment (Figure 1).

**Table 2:** Disinfection Efficacy for Monkeypox Virus (MPXV) on Six Surfaces^a^

**surface material**	**porosity**	**treatment**	**concentration**	**virus controlLog**_**10**_**(PFU/surf)**	**virus treatmentLog**_**10**_**(PFU/surface)**	**Log reduction**
glass	nonporous	dry wipe	NA	4.56 ± 0.28	3.6 ± 0.95	0.96 ± 0.99
		water wipe	NA		1.14 ± 1.02	3.41 ± 1.06
		NaClO	0.05%		ND	≥3.56
		NaClO	0.5%		ND	≥3.56
		ethanol	70%		ND	≥3.56
		H_2_O_2_	1.4%		0.63 ± 1.10	3.92 ± 1.13
		CHX, cetrimide	0.043%, 0.086%		ND	≥3.56
		BKZ	0.096%		ND	≥3.56
plastic	nonporous	dry wipe	NA	4.9 ± 0.28	4.78 ± 0.39	0.12 ± 0.48
		water wipe	NA		1.06 ± 0.92	3.84 ± 0.96
		NaClO	0.05%		ND	≥3.90
		NaClO	0.5%		ND	≥3.90
		Ethanol	70%		ND	≥3.90
		H_2_O_2_	1.4%		0.95 ± 1.64	3.95 ± 1.67
		CHX, cetrimide	0.043%, 0.086%		ND	≥3.90
		BKZ	0.096%		ND	≥3.90
steel	nonporous	dry wipe	NA	4.78 ± 0.48	4.35 ± 1.00	0.43 ± 1.11
		water wipe	NA		1.96 ± 0.65	2.94 ± 0.81
		NaClO	0.05%		ND	≥3.78
		NaClO	0.5%		ND	≥3.78
		ethanol	70%		ND	≥3.78
		H_2_O_2_	1.4%		1.81 ± 1.65	2.97 ± 1.72
		CHX, cetrimide	0.043%, 0.086%		ND	≥3.78
		BKZ	0.096%		ND	≥3.78
latex	NA	dry wipe	NA	4.53 ± 0.52	4.49 ± 0.16	0.04 ± 0.55
		water wipe	NA		2.69 ± 0.57	1.84 ± 0.77
		NaClO	0.05%		ND	≥3.53
		NaClO	0.5%		ND	≥3.53
		ethanol	70%		ND	≥3.53
		H_2_O_2_	1.4%		1.58 ± 0.34	2.95 ± 0.62
		CHX, Cetrimide	0.043%, 0.086%		ND	≥3.53
		BKZ	0.096%		ND	≥3.53
ceramic	porous	Dry wipe	NA	ND	ND	NA
		Water wipe	NA		ND	NA
		NaClO	0.05%		ND	NA
		NaClO	0.5%		ND	NA
		Ethanol	70%		ND	NA
		H2O2	1.4%		ND	NA
		CHX, Cetrimide	0.043%, 0.086%		ND	NA
		BKZ	0.096%		ND	NA
wood	porous	Dry wipe	NA	3.22 ± 0.26	3.16 ± 0.39	0.06 ± 0.47
		Water wipe	NA		2.98 ± 0.45	0.25 ± 0.52
		NaClO	0.05%		ND	≥2.22
		NaClO	0.5%		ND	≥2.22
		Ethanol	70%		ND	≥2.22
		H2O2	1.4%		2.54 ± 0.20	0.68 ± 0.32
		CHX, Cetrimide	0.043%, 0.086%		2.09 ± 0.60	1.14 ± 0.65
		BKZ	0.096%		1.46 ± 1.39	1.76 ± 1.42

a Titer of MPXV recovered after treatment (log_10_ PFU/surface) and logarithmic reduction values by surface material and treatment. Data represents the average and standard deviations of three independent replicates for each treatment. LOD of the assay was 10 PFU/surface. ND = non detected, NA = not applicable.

For porous surfaces, different disinfection patterns were observed (Figure 1, Table 2). For the ceramic coupons, we were not able to recover any of the inoculated MPXV in the control, dry wipe, or water wipe. Thus, disinfection efficacy was not able to be evaluated for ceramic. In contrast, on the wood coupon, the inoculated MPXV was partially absorbed to the surface, with a decline of ∼2 log_10_ on the virus titer control after inoculation. On wood, NaClO (0.05%, 0.5%) and ethanol reduced the MPXV to below the LOD (>2.22 log_10_ reduction). However, 1.4% H_2_O_2_, 0.096% BKZ, and 0.043% CHX with 0.086% cetrimide did not inactivate MPXV below the LOD, with inactivations of 0.7–1.8 log_10_, or 80–98.4%, reduction, depending on the disinfectant. For all the surfaces evaluated, porous and nonporous, NaClO at 0.5 and 0.05%, and 70% ethanol were able to reduce the inoculated MPXV below the limit of detection.

Our results demonstrate that 0.05% and 0.5% sodium hypochlorite solutions and 70% ethanol are efficacious against MPXV when wiped on common surfaces in low-resource settings with a 1 min contact time. Disinfectants containing QACs were efficacious on nonporous surfaces (≥3.5 log_10_ reduction or ≥99.97% inactivation), but had diminished efficacy on wood, a porous surface, underscoring the critical relationship between material porosity and specific disinfection methodology. Lastly, 1.4% H_2_O_2_ had only limited MPXV reduction across all tested surfaces. Below, we discuss the results by disinfectant, surface, and application method, and present limitations and recommendations for guidance and future research.

Chlorine-based disinfectants, including liquid sodium hypochlorite (NaClO), display a broad spectrum of antimicrobial activity and are effective against enveloped viruses at various concentrations.^41^ In this study, NaClO at a concentration of 0.05% was sufficient to achieve a complete inactivation of the inoculated MPXV (≥99.97% reduction for nonporous surfaces and ≥99.40% for porous surfaces) when evaluated at 1 min contact time. This is consistent with other studies on orthopoxviruses, which found 0.25% NaClO reduced vaccinia virus by >4 log_10_ on stainless-steel surfaces using a surface test method (disinfectant directly applied to the surface with no wiping) with 1 min contact time,^42^ and that 0.1% NaClO was one of the most effective of 20 disinfectants evaluated against variola virus using suspension tests.^25^ World Health Organization (WHO) and the U.S. Centers for Disease Control and Prevention (CDC) recommend 0.05%, 0.1%, and 0.5% for surface disinfection for other enveloped viruses (Ebola virus, SARS-CoV-2),^38,41^ and in emergency contexts recommended NaClO concentrations can be as high as 2%.^43^ Herein, we demonstrated that 0.05% NaClO concentrations, which are safer-to-use, particularly for health and care workers, and are less damaging to surfaces treated, are also efficacious against MPXV.

Generally, alcohols are broadly efficacious disinfectants against enveloped viruses.^44^ For orthopoxviruses, ethanol has been found to be efficacious against vaccinia virus in suspension tests in concentrations between 50 and 95% with 1 min contact time.^24^ Furthermore, suspension tests using MPXV have found 75% ethanol for 1 min contact time sufficient to achieve an >4 log_10_ inactivation.^26^ These are consistent with our results, which found that ethanol at 70% was sufficient to inactivate MPXV (>3.5 log_10_ reduction) on both nonporous and porous surfaces at 1 min contact time.

Overall, 1.4% H_2_O_2_ did not efficaciously reduce MPXV on surfaces below LOD, achieving reductions between 0.7 and 0.9 log_10_, depending on surface. This contrasts with two studies using suspension tests to evaluate H_2_O_2_ efficacy against vaccinia virus, which found 14.4% H_2_O_2_ inactivated vaccinia virus (>4 log_10_ reduction) with 30-s contact time,^45^ and 7.5% H_2_O_2_ achieved 4.9 log_10_ reduction in 10 min contact time.^42^ In our study, we evaluated a lower concentration (1.4%), which is commonly available in low-resource settings, for 1 min contact time, which is practical to complete. Our results align with other research, which has shown that in surface tests using stainless steel, alcohol-based disinfectants are more effective at inactivating MPXV than H_2_O_2_ solutions.^21^ Future studies could evaluate improved hydrogen peroxide products, which have been shown to be more effective than standard H_2_O_2_ at the same concentrations for inactivating healthcare associated pathogens, including MRSA (methicilin-resistant *Staphylococcus aureus*), VRE (vancomycin-resistant Enterococcus, and multidrug resistant Acinetobacter.^46^

In our results, two quaternary ammonium compounds reduced MPXV on nonporous surfaces by ≥3.5 log_10_ (≥99.97%). However, neither product reduced MPXV on porous wood below the LOD. The disinfectant containing BKZ achieved reductions of 1.8 log_10_ (98% reduction), while the disinfectant containing CHX and cetrimide achieved a 1.1 log_10_ (93%). Suspension tests evaluating the efficacy of BKZ-containing solutions at similar concentrations against vaccinia virus have shown incomplete inactivation with contact times between 1 and 10 min.^24,47,48^ Other quaternary ammonium compounds (QACs) exhibit diverse efficacy against orthopoxviruses,^24^ with several compounds incompletely inactivating the virus. In line with our results, solutions containing NaClO or 70% ethanol have more efficaciously inactivated orthopoxviruses (vaccinia virus) than QACs, including BKZ and benzyl dimethyl tetradecyl ammonium chloride.^24^

Surface type also impacted the inactivation. Nonporous surfaces such as plastic, glass, and metal allow for easy application and effective distribution of disinfectants, generally resulting in high disinfection efficacy. In contrast, porous surfaces (like wood) can absorb disinfectants, potentially reducing disinfectant efficacy.^37,39,49^ A previous systematic review on surface disinfection found disinfectants are less effective on porous or scratched surfaces, as compared to nonporous or smooth surfaces.^40^ In this study, we demonstrated MPXV was absorbed onto ceramic and wood coupons after inoculation. This absorption reduced the amount of recoverable virus and disinfectant efficacy. Previous studies have shown that the porosity and texture of the surface material influences virus persistence on surfaces^18,19,22^ and disinfection efficacy.^37,39,49,50^ Studies evaluating MPXV persistence on surfaces have shown that MPXV decays faster on porous surfaces.^18,19^ One explanation for this is that porous surfaces are more permeable, allowing liquids containing viruses to move through the material, thereby decreasing infectivity and recovery rate.^18,51^ Our findings underscore the importance of considering surface porosity when developing and applying disinfection protocols, as porous materials can compromise disinfectant efficacy. A key research question to answer is the relative risk of MPXV infection from nonporous and porous surfaces, to understand the relative importance of disinfecting nonporous and porous surfaces.

In addition to disinfectant and surface type, laboratory testing methodology also influences efficacy results. Suspension tests are commonly recommended to assess initial disinfectant efficacy, but their results may poorly predict real-world performance and tend to yield more favorable outcomes compared to surface coupon tests.^27−30,52^ Thus, coupon tests are a necessary second step to assess real-world efficacy. However, coupon tests are influenced by the mode of disinfectant application (e.g., spraying, wiping, immersion, inoculation on surface by pipetting), the mode of virus recovery from the surface, and the method used to neutralize the disinfectants.^39^ Even within the same application mode, high variation can occur. For example, studies evaluating disinfection efficacy using the spraying method have shown that outcomes vary based on the spraying equipment and parameters used, such as velocity, distance, and duration of spraying.^39,53^ In this study, we evaluated the efficacy of disinfectants using a wiping methodology designed to reflect real-world scenarios. This involved using a microfiber cloth saturated with disinfectant.^34,35^ Wiping also presents challenges, as variation can occur due to differences in the pressure applied, fabric, and wiping technique.^54^ To improve replicability of our results, we controlled the pressure applied. To replicate real-world conditions, we wiped only once over the surface. This provides a conservative estimate of wiping efficacy. Additionally, we found the mechanical action of wiping alone did inactivate some MPXV. Further research is recommended on MPXV inactivation with other, commonly used methods of disinfectant application, virus recovery, and disinfectant neutralization.

This study had limitations. As described above, we evaluated disinfection efficacy using only one methodology: one disinfectant application method, with one virus recovery and one disinfectant neutralization method, and testing only one concentration of BSA to simulate soiled conditions. The final concentration of BSA inoculated onto the coupon was 0.3 g/L (1:10 dilution of 3g/L BSA solution), which is considered low in various standard methods for assessing disinfectant efficacy. Many of these methods utilize higher concentrations of BSA, along with other proteins or animal blood, to simulate “dirty” conditions. A higher protein concentration could potentially reduce the effectiveness of disinfectants. Therefore, future studies should assess disinfectant efficacy under higher soil loads. In addition to these limitations, the maximum log_10_ inactivation we could observe was 3.5–3.9, due to our initial MPXV inoculum concentration of 5 × 10^7^ PFU/mL. Therefore, the actual MPXV inactivation could be higher than reported here. Moreover, we did not control for temperature and humidity during the experiments, which can influence disinfectant efficacy.^55−57^ Lastly, we evaluated a limited number of disinfectants and surfaces, most at a single concentration, all with a 1 min contact time. Higher concentrations and longer contact times could have resulted in higher MPXV inactivation, although they might not be practical.

Future studies should be conducted to expand upon these results, including with additional disinfectant application methods (wiping more or with other fabrics, varying pressure), virus recovery methods, disinfectant neutralization methods, higher initial inoculation concentrations, under environmental conditions (temperature, humidity) relevant to mpox endemic areas, and with more real-world surfaces and disinfectants. Additionally, as porous surfaces are more challenging to disinfect, further research is needed to understand the relationships between surface type (nonporous/porous), adsorption into the surface, disinfection efficacy, and transmission risk. Lastly, research is needed on surface-adjacent research, such as disinfectant efficacy in laundering and handwashing practices.

Studies quantifying MPXV on surfaces in contact with mpox patients have reported concentrations as high as 3.2 × 10^2^ PFU/sample.^15^ Our findings indicate that sodium hypochlorite solutions and ethanol would completely inactivate MPXV at that concentration, and even 1 order of magnitude higher. QACs would inactive MPXV on nonporous surfaces only, and H_2_O_2_ would not inactivate MPXV at the concentration tested (1.4%). Based on these results, we currently recommend using 0.05% sodium hypochlorite solutions or 70% ethanol for 1 min contact time to inactive MPXV on clean nonporous and porous surfaces.
